# Blends of Poly(3-Hydroxybutyrate-*co*-3-Hydroxyvalerate) with Fruit Pulp Biowaste Derived Poly(3-Hydroxybutyrate-*co*-3-Hydroxyvalerate-*co*-3-Hydroxyhexanoate) for Organic Recycling Food Packaging

**DOI:** 10.3390/polym13071155

**Published:** 2021-04-04

**Authors:** Beatriz Meléndez-Rodríguez, Sergio Torres-Giner, Maria A. M. Reis, Fernando Silva, Mariana Matos, Luis Cabedo, José María Lagarón

**Affiliations:** 1 Novel Materials and Nanotechnology Group, Institute of Agrochemistry and Food Technology (IATA), Spanish Council for Scientific Research (CSIC), Calle Catedrático Agustín Escardino Benlloch 7, 46980 Paterna, Spain; beatriz.melendez@iata.csic.es (B.M.-R.); storresginer@iata.csic.es (S.T.-G.); 2 UCIBIO-REQUIMTE-Applied Molecular Biosciences Unit, Chemistry Department, Faculty of Sciences and Technology, New University of Lisbon, 1099-085 Lisbon, Portugal; amr@fct.unl.pt (M.A.M.R.); fra.silva@campus.fct.unl.pt (F.S.); m.matos@campus.fct.unl.pt (M.M.); 3 Polymers and Advanced Materials Group (PIMA), Universitat Jaume I (UJI), 12071 Castellón, Spain; lcabedo@uji.es

**Keywords:** polyhydroxyalkanoates, waste valorization, food packaging, Circular Bioeconomy, organic recycling

## Abstract

In the present study, a new poly(3-hydroxybutyrate-*co*-3-hydroxyvalerate-*co*-3-hydroxyhexanoate) [P(3HB-*co*-3HV-*co*-3HHx)] terpolyester with approximately 68 mol% of 3-hydroxybutyrate (3HB), 17 mol% of 3-hydroxyvalerate (3HV), and 15 mol% of 3-hydroxyhexanoate (3HHx) was obtained via the mixed microbial culture (MMC) technology using fruit pulps as feedstock, a processing by-product of the juice industry. After extraction and purification performed in a single step, the P(3HB-*co*-3HV-*co*-3HHx) powder was melt-mixed, for the first time, in contents of 10, 25, and 50 wt% with commercial poly(3-hydroxybutyrate-*co*-3-hydroxyvalerate) (PHBV). Thereafter, the resultant doughs were thermo-compressed to obtain highly miscible films with good optical properties, which can be of interest in rigid and semirigid organic recyclable food packaging applications. The results showed that the developed blends exhibited a progressively lower melting enthalpy with increasing the incorporation of P(3HB-*co*-3HV-*co*-3HHx), but retained the PHB crystalline morphology, albeit with an inferred lower crystalline density. Moreover, all the melt-mixed blends were thermally stable up to nearly 240 °C. As the content of terpolymer increased in the blends, the mechanical response of their films showed a brittle-to-ductile transition. On the other hand, the permeabilities to water vapor, oxygen, and, more notably, limonene were seen to increase. On the overall, this study demonstrates the value of using industrial biowaste derived P(3HB-*co*-3HV-*co*-3HHx) terpolyesters as potentially cost-effective and sustainable plasticizing additives to balance the physical properties of organic recyclable polyhydroxyalkanoate (PHA)-based food packaging materials.

## 1. Introduction

The massive use of synthetic plastics in recent decades has caused a deep damage to the environment. Polymers derived from fossil hydrocarbons are habitually non-biodegradable, making them difficult to eliminate and being accumulated in landfills or in natural environments [1]. This environmental issue becomes particularly relevant for packaging applications, in which the articles are generally discarded after a short and single use and, thus, a change in the strategies related to the type of material used is necessary [2,3].

Polyhydroxyalkanoates (PHAs) are a family of linear polyesters produced in nature by the action of bacteria, both Gram-positives and Gram-negatives, during fermentation of sugar or lipids in famine conditions [4]. PHAs are currently regarded as a good alternative to conventional petroleum-based polymers. These biopolyesters are biodegradable both in composting conditions and in natural environments, while they are fully bio-based. Therefore, PHAs can be integrated inside the so-called Circular Bioeconomy strategies that aim, by organic recycling, to bring the carbon back to the soil [5]. The first identified and best-characterized PHA was poly(3-hydroxybutyrate) (PHB) [6]. However, PHB has poor physical properties in terms of brittleness and low processability due to its highly crystalline nature [7]. The use of PHA copolymers can improve these properties and the most common one is poly(3-hydroxybutyrate-*co*-3-hydroxyvalerate) (PHBV). In particular, the percentage of 3-hydroxyvalerate (3HV) in the copolyester can modify the relevant properties of the polymer. For example, the degree of crystallinity of PHB can decrease up to approximately 40% with increasing the 3HV content to 30 mol% that, in turns, reduces the melting temperature (T_m_) and brittleness while improves the processing window [8]. Similarly, the mechanical properties are enhanced in terms of ductility when the 3HV content increases [9]. Thus, an increased in elongation at break (ε_b_) from 5.6%, for PHB, to 690%, was reported for a film of PHBV with 20 mol% 3HV prepared by solvent casting [10]. In another study, ε_b_ increased from 3% to 44% in solvent-casted PHBV films with a change in the 3HV content from 7 mol% to 40 mol% [11]. Furthermore, the use of terpolymers has also been identified as an effective strategy to improve the material properties of PHB [12]. In this regard, the poly(3-hydroxybutyrate-*co*-3-hydroxyvalerate-*co*-3-hydroxyhexanoate) [P(3HB-*co*-3HV-*co*-3HHx) or PHBVHHx] terpolyester is very promising. For instance, Zhao et al. [12] produced various P(3HB-*co*-3HV-*co*-3HHx) terpolymers with 3HV and 3-hydroxyhexanoate (3HHx) molar contents ranging between 2.3–7.1 mol% and 5.0–15.1 mol%, respectively. It was demonstrated that the P(3HB-*co*-3HV-*co*-3HHx) terpolymer shows higher thermal stability and ε_b_ compared to the PHB homopolymer and its PHBV copolymer with 5 mol% 3HV and also poly(3-hydroxybutyrate-*co*-3-hydroxyhexanoate) [P(3HB-*co*-3HHx)] with 12 mol% 3HHx. In particular, ε_b_ increased from 4.5%, for PHB, to 481.1% for P(3HB-*co*-3HV-*co*-3HHx) with 13.4 mol% 3HHx content. P(3HB-*co*-3HV-*co*-3HHx) also presented lower melting temperatures and enthalpies of fusion than the homopolymer, that is, 162 °C and 97 J/g, for PHB, and 104 °C and 20.8 J/g, for the terpolyester with 15.1 mol% 3HHx content, respectively. This fact is due to its lower crystallinity and the formation of crystals with lower degree of perfection. Moreover, Ye et al. [13] reported that P(3HB-*co*-3HV-*co*-3HHx) showed higher crystallization rate and degree of crystallinity than P(3HB-*co*-3HHx), and also an improvement of the ductile performance, which was explained by the simultaneous introduction of 3HV and 3HHx monomers. Thus, one can consider that all these changes in the material properties would positively contribute to attain property-balanced materials, more suitable for use in food packaging. Furthermore, some studies have indicated that these terpolymers are biocompatible and non-cytotoxic [14,15].

Nevertheless, the synthesis of P(3HB-*co*-3HV-*co*-3HHx) has been scarcely reported in a few studies. Some bacterial strains have been used in the P(3HB-*co*-3HV-*co*-3HHx) production, such as *Rhodospirillum rubrum* and *Rhodococcus* sp. NCIMB 40,126 [16,17]. Moreover, the use of recombinant strains modified through genetic engineering has been described to obtain a higher control of the resultant PHA, such as *Escherichia coli* [18], *Cupriavidus necator* [19], *Ralstonia eutropha* [20], among others [21,22]. However, the use of pure microbial cultures for PHA production requires pure substrates as the carbon resources and also sterile conditions, making this process expensive and not highly sustainable from an industrial upscaling point of view [23]. An alternative to reduce the market price while increasing sustainability would be through the use of mixed microbial cultures (MMCs) in combination with the use of industrial by-products of agro-food wastes such as feedstocks [24,25]. In this regard, there are several organic wastes of industrial by-products that have been reported for the production of PHAs such as oil mill [26,27], molasses [28], paper mill effluents [29], dairy whey [30,31], fermented fruit [32,33], and municipal solid waste [34]. In this sense, the use of biowastes for PHA production has shown to increase the economic value of the final product, while it reduces the overall environmental impact of the biopolymer production [35].

Blending by melt mixing with additives and fillers has also demonstrated to be a convenient approach to improve the properties of PHAs. Thus, it is common to use mixtures with different commercial biopolymers [36,37], waste derived fillers [38,39] or a combination of both [40,41], which increase some of the final properties such as thermal, mechanical or barrier. These properties can be modified depending on the polymers and the ratios used in the blends [42]. The techniques most commonly used to prepare the PHA blends are solvent solution [43,44] and melt mixing [45,46]. The latter technique shows certain advantages as it is a fast method, it is environmentally friendly as no solvents are required, and large mass production can be easily scaled up [47,48]. In this context, the use of waste derived PHA in melt-mixed blends with commercial PHA has been previously assessed by our group. Thus, Martinez-Abad et al. [49] reported blends of commercial PHBV with unpurified PHBV obtained by MMCs from cheese whey (CW). The obtained blends showed good miscibility and their thermal, mechanical, and barrier properties were not substantially affected for loadings of up to 10 wt% of the food waste derived PHBV, whereas the sustainable profile of PHBV was improved. Also, blends of commercial PHB and purified PHBV obtained from fruit pulp waste filled with 10 wt% rice husk flour (RHF) also showed good miscibility, increased thermal stability, and slightly better mechanical properties in terms of strength and ductility [40].

In this study, a newly developed P(3HB-*co*-3HV-*co*-3HHx) produced by MMC using biomass derived from fruit pulp, an industrial by-product of the juice industry, was melt-mixed with commercial PHBV at contents from 10 to 50 wt%. The neat PHAs and resultant PHBV/P(3HB-*co*-3HV-*co*-3HHx) blends were subsequently thermo-compressed to produce films that were characterized in terms of their morphology and optical characteristics as well as thermal, mechanical, and barrier properties to evaluate their potential in food packaging applications.

## 2. Materials and Methods

### 2.1. Materials

A commercial PHBV grade, ENMAT^TM^ Y1000P, was obtained in the form of pellets from Tianan Biologic Materials (Ningbo, China). According to the manufacturer, the 3HV fraction in the copolyester is 2 mol% and the weight-average molecular weight (M_W_) is ~2.8 × 10^5^ g/mol. P(3HB-*co*-3HV-*co*-3HHx) terpolyester was produced at pilot-plant scale at Universidade NOVA (Lisboa, Portugal) using the technology of MMC fed with fermented fruit pulp supplied by Sumol+Compal S.A. (Carnaxide, Portugal), as an industrial residue of the juice industry.

_D_-limonene, with 98% purity, was purchased from Sigma-Aldrich S.A. (Madrid, Spain). Chloroform, stabilized with ethanol and 99.8% purity, and sodium hydroxide (NaOH) were obtained from Panreac S.A. (Barcelona, Spain). Methyl 3-hydroxyhexanoate and heptadecane were supplied by Sigma Aldrich Química S.A. (Sintra, Portugal).

### 2.2. Production of P(3HB-co-3HV-co-3HHx)

The experimental setup to produce a terpolymer of P(3HB-*co*-3HV-*co*-3HHx) consisted of 3-pilot scale bioreactors inoculated with biomass from wastewater treatment plants for the microorganism selection. A 60-L up-flow anaerobic sludge blanket (UASB) reactor fed with fruit pulp waste was used to produce the precursors for PHA. This reactor was operated at hydraulic retention time (HRT) of 1 day, a temperature of 30 °C, and the pH was kept at approximately 5.0. After filtration, the effluent rich in fermentation products (FP) was fed as carbon source to the subsequent steps of the process. A sequencing batch reactor (SBR) with a volume of 100 L was fed with the FP and operated under a feast and famine regime with the goal of producing a PHA-accumulating culture. The reactor operated in 12-h cycles consisting of 11 h of aeration and 1 h of settling. The HRT and sludge retention time were set to 1 day and 4 days, respectively. PHA accumulation was carried out in a 50-L aerobic fed-batch reactor inoculated with 25 L of biomass purged from the SBR at the end of famine phase. The reactor was fed with the FP-rich effluent from the UASB reactor in a pulse-wise mode controlled by the dissolved oxygen (DO) response.

A mass with a P(3HB-*co*-3HV-*co*-3HHx) content of approximately 41 wt% was attained at the end of the accumulation assay. The terpolymer showed the following composition: 68 mol% of 3-hydroxybutyrate (3HB), 17 mol% of 3HV, and 15 mol% of 3HHx. Results were obtained by gas chromatography (GC) according to the methodology described by Lanham et al. [50]. Samples were calibrated through standard curves with a solution made of a commercial copolymer of PHBV with 12 mol% 3HV content and methyl 3-hydroxyhexanoate and heptadecane as internal standard. The M_W_ of the P(3HB-*co*-3HV-*co*-3HHx) was 7.90 × 10^5^ g/mol and the PDI 1.62, which was measured by size exclusion chromatography (SEC) as described by Rebocho et al. [51].

Finally, the medium-containing PHA was lyophilized. For this, the liquid material was first neutralized with a 2M solution of NaOH and then centrifuged three consecutive times at 4000 rpm for 15 min. The resultant pellet was washed gently with distilled water. The obtained material was stored at −80 °C for at least 3 h and freeze-dried for a week to produce an unpurified biomass powder containing the P(3HB-*co*-3HV-*co*-3HHx).

### 2.3. Extraction and Purification of P(3HB-co-3HV-co-3HHx)

The unpurified biomass containing the P(3HB-*co*-3HV-*co*-3HHx) was processed following the previously reported chloroform-based extraction and purification one-step method [52]. For this, the biomass was dissolved in chloroform at 5 wt% and the mixture was then stirred for 24 h at 50 °C to degrade the non-PHA cellular material. Later, the solution was transferred to centrifugation tubes in which distilled water was added at 50 wt%. After shaking the tubes manually, these were centrifuged for 5 min at 4000 rpm in an Avanti J-26S XP Centrifuge with a JLA-16.250 Rotor (maximum radius: 134 mm; average radius: 90 mm; minimum radius: 46 mm, Beckman Coulter, CA, USA). Afterwards, the P(3HB-*co*-3HV-*co*-3HHx) phase was recovered from the bottom of the tubes with a pipette and transferred to beakers, leaving them in the extractor hood until the solvent was completely evaporated.

### 2.4. Melt Mixing

Prior to processing, both PHA resins were vacuum-dried at 60 °C for 24 h in an oven (Digitheat, JP Selecta S.A., Barcelona, Spain) to remove any residual moisture. Then, different amounts of the purified P(3HB-*co*-3HV-*co*-3HHx) powder, from 10 to 50 wt%, were manually pre-mixed with the commercial PHBV pellets in a zipper bag. Neat PHBV and P(3HB-*co*-3HV-*co*-3HHx) formulations were also prepared under identical processing conditions as control materials. Table 1 summarizes the different formulations prepared.

To prepare each sample, a total amount of 12 g of material was melt-compounded in a 16-cm^3^ Brabender Plastograph Original E internal mixer from Brabender GmbH & Co. KG (Duisburg, Germany). The mixture was fed to the internal mixing chamber at a rotating speed of 60 rpm for 1 min and, after this, it was melt-mixed at 100 rpm for another 3 min. The processing temperature was set at 180 °C. Once the mixing process was completed, each batch was withdrawn from the mini-mixer and allowed to cool at room temperature. The resultant doughs were stored in dissectors containing silica gel at 0% relative humidity (RH) and 25 °C for, at least, 48 h for conditioning.

The different doughs were, thereafter, thermo-compressed into films using a hot-plate hydraulic press (Carver 4122, Wabash, IN, USA). The samples were first placed in the plates at 180 °C for 1 min, without pressure, to ensure thermal softening of the biopolymers and then hot-pressed at 4–5 bars for 3 min. In the case of the neat P(3HB-*co*-3HV-*co*-3HHx), the temperature was reduced to 155 °C since this was the lowest molding temperature in which, after the intensive melt-mixing step, the pure material did not show signs of degradation. Flat films of 10 cm × 10 cm with a total thickness of ca. 100 µm were obtained and stored in a desiccator at 25 °C and 0% RH for 15 days prior to characterization.

### 2.5. Characterization

#### 2.5.1. Scanning Electron Microscopy

The morphologies of the film cross-sections were observed by scanning electron microscopy (SEM) using an S-4800 device from Hitachi (Tokyo, Japan). For the cross-section observations, the films were cryo-fractured by immersion in liquid nitrogen. Sample observations were performed following the conditions reported previously [40]. The estimation of the dimensions was performed by means of the ImageJ software v 1.41 (National Institutes of Health, Bethesda, Maryland, USA) using a minimum of 20 SEM micrographs.

#### 2.5.2. Transparency

The light transmission of the films was determined in specimens of 50 mm × 30 mm by quantifying the absorption of light at wavelengths between 200 and 700 nm in an ultraviolet– visible (UV–Vis) spectrophotometer VIS3000 from Dinko Instruments (Barcelona, Spain). The transparency (T) and opacity (O) were calculated using Equation (1) [53] and Equation (2) [54], respectively:

(1) $$\;\;\;\;\;T=\frac{A_{600}}{L}$$

(2) $$O=A_{500}\times L$$

where A_500_ and A_600_ are the absorbance values at 500 and 600 nm, respectively, and L is the film thickness (mm).

#### 2.5.3. Color Measurements

The color of the films was determined using a chroma meter CR-400 (Konica Minolta, Tokyo, Japan). The color difference (∆E*) was calculated using the following Equation (3) [55], as defined by the Commission Internationale de l’Eclairage (CIE):

(3) $$\Delta E^{*\;}={\left[{\left(\Delta L^{*}\right)}^{2}+{\left(\Delta a^{*}\right)}^{2}+{\left(\Delta b^{*}\right)}^{2}\right]}^{0.5}$$

where ∆L*, ∆a*, and ∆b* correspond to the differences in terms of lightness from black to white, color from green to red, and color from blue to yellow, respectively, between the test samples and the control sample of commercial PHBV. Color change was evaluated using the following assessment [56]: Unnoticeable (ΔE* < 1), only an experienced observer can notice the difference (ΔE* ≥ 1 and < 2), an unexperienced observer notices the difference (ΔE* ≥ 2 and < 3.5), clear noticeable difference (ΔE* ≥ 3.5 and < 5), and the observer notices different colors (ΔE* ≥ 5).

#### 2.5.4. Thermal Analysis

Thermal transitions of the films were studied by differential scanning calorimetry (DSC) on a DSC 8000 device from PerkinElmer, Inc. (Waltham, MA, USA), equipped with the cooling accessory Intracooler 2 from PerkinElmer, Inc. A three-step program under nitrogen atmosphere and with a flow-rate of 20 mL/min was applied: First heating from −30 to 180 °C, followed by cooling to −30 °C, and completed by a second heating to 200 °C. The heating and cooling rates were both set at 10 °C/min and the typical sample weight was ~3 mg. An empty aluminum pan was used as reference, whereas calibration was performed using an indium sample. The glass transition temperature (T_g_), cold crystallization temperature (T_cc_), enthalpy of cold crystallization (ΔH_cc_), T_m_, and enthalpy of melting (ΔH_m_) were obtained from the heating scans, while the crystallization temperature from the melt (T_c_) and enthalpy of crystallization (ΔH_c_) were determined from the cooling scan. All DSC measurements were performed in triplicate.

Thermogravimetric analysis (TGA) was performed in a TGA-550 device (TA Instruments, New Castle, DE, USA). Film samples, with a weight of ~15 mg, were heated from 50 to 800 °C at a heating rate of 10 °C/min under a flow-rate of 50 mL/min of nitrogen (N_2_). All TGA measurements were done in triplicate.

#### 2.5.5. WAXD Experiments

Wide angle X-ray diffraction (WAXD) measurements were performed using a Bruker AXS D4 ENDEAVOR diffractometer (Billerica, MA, USA). The samples were scanned, at room temperature, in the reflection mode using incident Cu K-alpha (α) radiation (k = 1.54 Å), while the generator was set up at 40 kV and 40 mA. The data were collected over the range of scattering angles (2θ) comprised in the 2–40° range.

#### 2.5.6. Mechanical Tests

Tensile tests were performed according to the ASTM D638 standard, using dumbbell samples (Type IV) die-cut from the hot-pressed films. Tensile tests were conducted in a universal testing machine (Shimatzu AGS-X 500N) at room temperature with a cross-head speed of 10 mm/min. Samples were conditioned for 24 h prior to analysis and the tests were performed at room conditions, that is, 40% RH and 25 °C. A minimum of six specimens were tested for each sample.

#### 2.5.7. Permeability Tests

The water vapor permeability (WVP) and _D_-limonene permeability (LP) of the films were determined following the standardized gravimetric method ASTM E96-95. To do this, Payne permeability cups of 3.5 cm from Elcometer Sprl (Hermallesous-Argenteau, Belgium) were used. Both tests were performed at 25 °C in triplicate, and further details can be found elsewhere [40].

The oxygen permeability coefficient was derived from the oxygen transmission rate (OTR) measurements that were recorded at 60% RH and 25 °C, in duplicate, using an Oxygen Permeation Analyzer M8001 (Systech Illinois, Thame, UK). The humidity equilibrated samples were purged with nitrogen, before exposure to an oxygen flow of 10 mL/min. The exposure area during the test was 5 cm^2^ for each sample. In order to obtain the oxygen permeability (OP), film thickness and gas partial pressure were considered.

### 2.6. Statistical Analysis

The optical, thermal, mechanical, and barrier properties were evaluated through analysis of variance (ANOVA) using STATGRAPHICS Centurion XVI v 16.1.03 from StatPoint Technologies, Inc. (Warrenton, VA, USA). Fisher’s least significant difference (LSD) was used at the 95% confidence level (*p* < 0.05). Mean values and standard deviations were also reported.

## 3. Results and Discussion

### 3.1. Morphology

**Figure 1:**
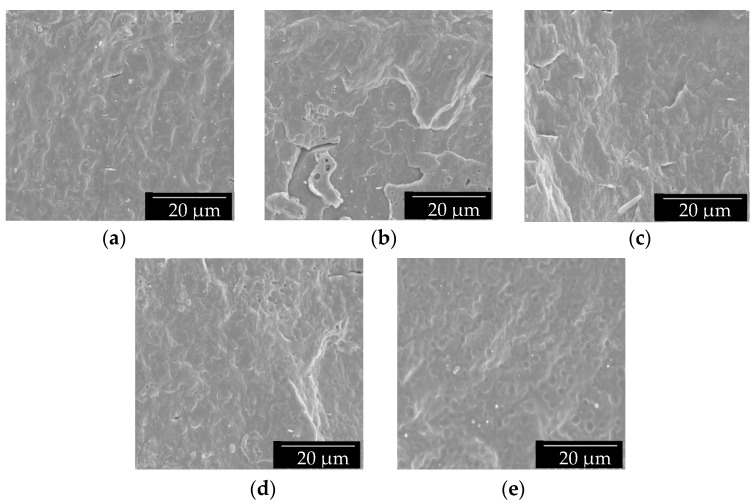
Scanning electron microscopy (SEM) images of the cross-sections of the thermo-compressed films made of poly(3-hydroxybutyrate-*co*-3-hydroxyvalerate) (PHBV) and poly(3-hydroxybutyrate-*co*-3-hydroxyvalerate-*co*-3-hydroxyhexanoate) [P(3HB-*co*-3HV-*co*-3HHx)]: (**a**) PHBV; (**b**) PHBV90/P(3HB-*co*-3HV-*co*-3HHx)10; (**c**) PHBV75/P(3HB-*co*-3HV-*co*-3HHx)25; (**d**) PHBV50/P(3HB-*co*-3HV-*co*-3HHx)50; (**e**) P(3HB-*co*-3HV-*co*-3HHx). Images were taken at 2000x with scale markers of 20 µm.

The morphology of the film cross-sections was observed by SEM and the images are gathered in Figure 1. It can be observed that all the films presented a smooth and featureless fracture surface, without significant plastic deformation, indicating a typical brittle fracture behavior. Moreover, both good mixture and compatibility between the two PHAs were expected to be attained since no indications of phase segregation were observed, even at the highest P(3HB-*co*-3HV-*co*-3HHx) content, that is, 50 wt%. However, the film fracture surfaces of the PHBV-based materials revealed the presence of some microparticles, which can be ascribed to nucleating agents added by the manufacturer, such as boron nitride [57]. In the case of the neat P(3HB-*co*-3HV-*co*-3HHx), one can observe the presence of some impurities in the form of small lumps, which can be related to organic traces of cell debris or fatty acids derived from the bioproduction process of P(3HB-*co*-3HV-*co*-3HHx). Similar observations were previously reported in blends made of commercial PHB and food waste derived PHBV [40]. Also, Martinez-Abad et al. [49] observed, for PHBV/unpurified PHBV blends, a good degree of interaction between the two PHAs with small amounts of impurities related to the biowaste polymer. Therefore, it can be concluded that both the commercial PHBV and the food waste derived P(3HB-*co*-3HV-*co*-3HHx) presented good miscibility since both biopolymers share a majority of 3HB content.

### 3.2. Optical Properties

**Figure 2:**
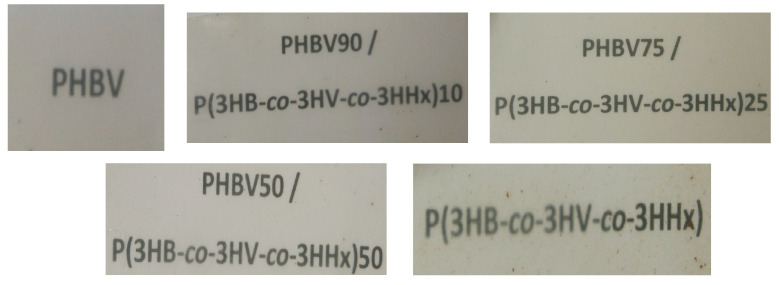
Contact transparency images of the thermo-compressed films of poly(3-hydroxybutyrate-*co*-3-hydroxyvalerate) (PHBV), poly(3-hydroxybutyrate-*co*-3-hydroxyvalerate-*co*-3-hydroxyhexanoate) [P(3HB-*co*-3HV-*co*-3HHx)], and their blends.

Figure 2 displays the visual aspect of the films to assess their optical properties. Simple naked eye examination of this figure indicates that all the film samples were slightly opaque, but they also showed good contact transparency with a brownish yellow color. For the film samples with the highest concentrations in P(3HB-*co*-3HV-*co*-3HHx), that is, 50 wt% and, especially, for the neat terpolymer film, small brown lumps can be observed in the material. These structures can be related to small impurities of the fruit juice based on cellulose that remained after the purification process and could develop a somewhat darker color due to processing. This result points out that additional efforts in the development of alternative more efficient purification processes will be required in the future for the optimal performance of these biopolymers.

To quantify the color change resulting from the addition of P(3HB-*co*-3HV-*co*-3HHx) to PHBV, the color coordinates (a*, b*, L*) and the values of ΔE*, T, and O were determined and reported in Table 2. From this, it can be observed that all the film samples presented similar values for the a*, b* and L* coordinates, with a tendency to green and yellow color, confirming the brownish color of the blends. The incorporation of P(3HB-*co*-3HV-*co*-3HHx) into the films slightly altered the color properties, being the differences still significant. However, the L* values were not significantly different except for the neat P(3HB-*co*-3HV-*co*-3HHx) film, which was the darkest sample. In any case, the color differences in the composite films containing different amounts of P(3HB-*co*-3HV-*co*-3HHx) with respect to the neat PHBV were relatively low, that is, ΔE* ≥ 1 and < 2, which means that only an experienced observer could notice the difference. As opposite, the neat P(3HB-*co*-3HV-*co*-3HHx) film presented a higher AE* value, near to 5, hence, there was a clear noticeable difference with the control sample. It can also be noticed that all the blend films presented similar transparency and opacity that the neat PHBV, except the pure P(3HB-*co*-3HV-*co*-3HHx), which presented the highest transparency with a value of approximately 2.9. The latter value can be related to a lower degree of crystallinity, as it will be discussed below. Although light colors may seem an advantage for use in packaging, slightly brownish color in the films may act as an UV barrier to prevent light-induced lipids oxidation, which is also important in food applications [58].

### 3.3. Thermal Properties

**Figure 3:**
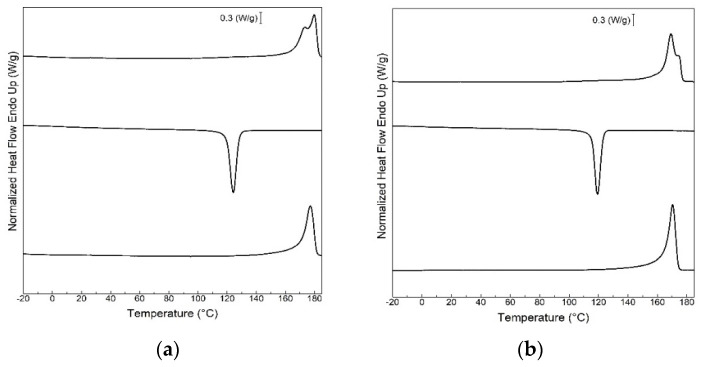

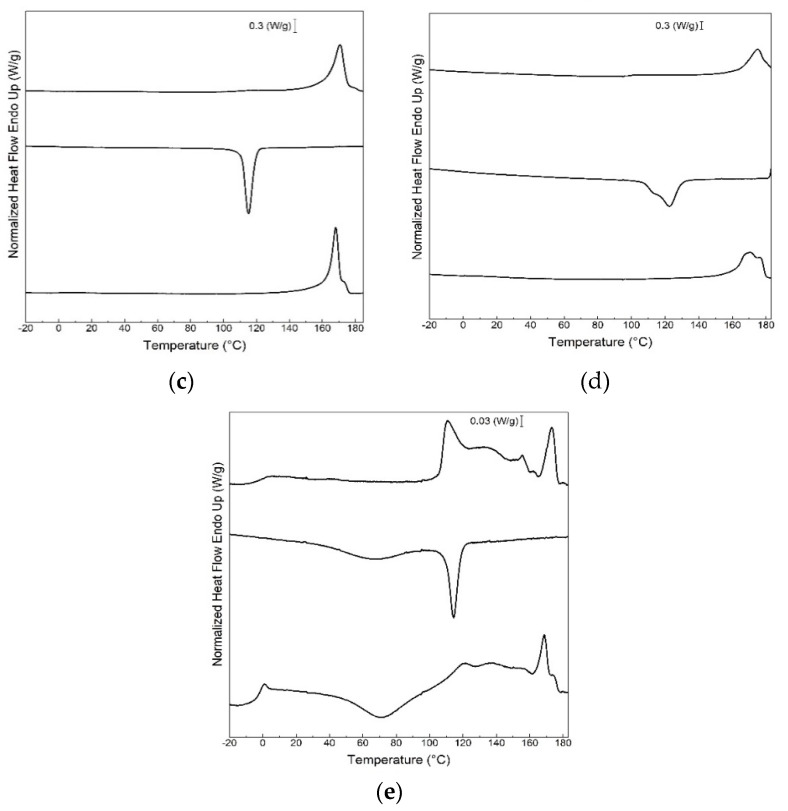
Differential scanning calorimetry (DSC) curves during first heating, cooling, and second heating of the thermo-compressed films of: (**a**) Neat poly(3-hydroxybutyrate-*co*-3-hydroxyvalerate) (PHBV); (**b**) PHBV90/P(3HB-*co*-3HV-*co*-3HHx)10; (**c**) PHBV75/P(3HB-*co*-3HV-*co*-3HHx)25; (**d**) PHBV50/P(3HB-*co*-3HV-*co*-3HHx)50; (**e**) neat poly(3-hydroxybutyrate-*co*-3-hydroxyvalerate-*co*-3-hydroxyhexanoate) [P(3HB-*co*-3HV-*co*-3HHx)].

Figure 3 displays the DSC thermograms of the film samples obtained during the first heating, cooling, and second heating. The summary of the main thermal transitions obtained from the DSC curves are gathered in Table S1, which is available in the Supporting Information. One can observe that the film samples showed a glass transition region with T_g_ at approximately 1.7 and 0.2 °C for the neat PHBV and P(3HB-*co*-3HV-*co*-3HHx) films, respectively, whereas intermediate values in the range of 1.4–0.6 °C were attained for their blends, suggesting intercomponent miscibility. During the first heating, the neat PHBV presented an endothermic peak with two components, with maximum at 178 °C and a ∆H_m_ value of 74 J/g. The blends with different contents in P(3HB-*co*-3HV-*co*-3HHx) presented the maximum of melting in the range of 169–174 °C with ∆H_m_ values of 74 and 68 J/g for the film samples with 10 and 25 wt% of terpolymer, respectively, whereas the films with 50 wt% of terpolymer showed a value of 43 J/g. As it can also be observed in the DSC curves, the neat P(3HB-*co*-3HV-*co*-3HHx) presented a very complex melting behavior, exhibiting the highest endothermic peaks at 111 °C, ascribed to the lowest crystalline density fractions of the terpolymer [59,60], and at 173 °C, for the most thermodynamically stable 3HB-rich fractions [61]; and having a whole ∆H_m_ value of 33 J/g. Thus, in the blend samples, a drop in T_m_ and ∆H_m_ was generally observed when P(3HB-*co*-3HV-*co*-3HHx) was incorporated into PHBV.

Regarding the cooling step, the samples with terpolymer contents up to 25 wt% crystallized from the melt in a single peak. However, for higher contents and, especially for the pure terpolymer, multiple broader crystallization events were observed. The neat PHBV showed a T_c_ at 123 °C, while their blends with P(3HB-*co*-3HV-*co*-3HHx) exhibited less intense T_c_ peaks that also shifted to lower values, in the 115–119 °C range. In the case of the neat P(3HB-*co*-3HV-*co*-3HHx), this film sample showed two clear crystallization peaks. The first one, which is ascribed to the 3HB-rich crystalline fractions [62,63], appeared in the form of a sharp peak at 114 °C and the other, as a broader peak and at the lower temperature of 65 °C, being ascribed to other crystalline fractions within the terpolymer requiring higher undercooling to crystallize. Moreover, the neat P(3HB-*co*-3HV-*co*-3HHx) showed a clear exothermic cold crystallization event during the second heating step, with T_cc_ at approximately 72 °C. In the second heating run, the blend samples were seen to present a reduction in ∆H_m_. In particular, whereas the neat PHBV showed a value of 85 J/g, the blends presented values of 76, 66, and 51 J/g for terpolymer contents of 10, 25 and 50 wt%, respectively. In the case of the neat P(3HB-*co*-3HV-*co*-3HHx), it showed the lowest ∆H_m_, having a value of 24 J/g, which confirms the lowest and most ill-defined crystallinity for this sample. The reduction in crystallinity, inferred from the lower ∆H_m_, in the terpolymer can be attributed to the other two terpolymer fractions impairing the crystallization of the 3HB fractions [12]. Since both PHAs were highly miscible, this factor may add further disturbance to the molecular lateral order of the terpolymer. In this regard, Kai et al. [64] reported a decrease in the crystallization degree and crystallization rate of PHB when P(3HB-*co*-3HHx) was introduced into the blends. Qu et al. [65] also showed a decrease in crystallinity with increasing 3HHx content in the P(3HB-*co*-3HHx) copolyester due to the diluting effect of the 3HHx component on the crystallinity of the 3HB parts. Furthermore, a decrease in crystallinity has been previously reported for other miscible polymer systems. For example, polylactide (PLA) melt-compounded with oligomer of lactic acid (OLA) also showed a reduction in the T_m_ values with respect to the neat PLA [66]. In particular, the oligomer impaired crystallization by inhibiting the correct packing of the PLA chains, although it also enhanced its mobility due to a plasticizing effect.

### 3.4. Crystallinity

**Figure 4:**
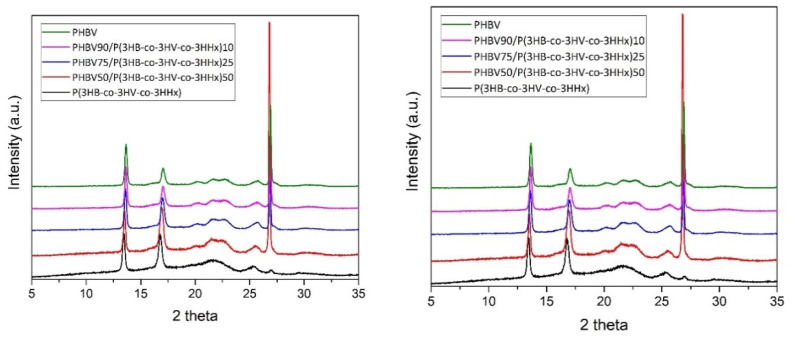
Wide angle X-ray diffraction (WAXD) patterns in the 2theta (2θ) ranges 5–35° (**left**) and 12–18.5° (**right**), from top to bottom, of the thermo-compressed films of: Neat poly(3-hydroxybutyrate-*co*-3-hydroxyvalerate) (PHBV); PHBV90/P(3HB-*co*-3HV-*co*-3HHx)10; PHBV75/P(3HB-*co*-3HV-*co*-3HHx)25; PHBV50/P(3HB-*co*-3HV-*co*-3HHx)50; and neat poly(3-hydroxybutyrate-*co*-3-hydroxyvalerate-*co*-3-hydroxyhexanoate) [P(3HB-*co*-3HV-*co*-3HHx)]. The data was normalized to the intensity of the (020) peak and shifted along the *Y*-axis for comparison purposes.

The crystalline phase was also studied by WAXD experiments on the PHA films. The diffractograms, included in Figure 4, revealed two main peaks located at 13.6 and 17.0° 2θ, corresponding to the (020) and (110) lattice planes of the orthorhombic unit cell of PHB [67]. These intense peaks were followed by three other minor reflections centered at approximately 22.6, 25.8, and 26.9° 2θ, which originate from (111), (121), and (040) lattice planes [61,68]. The diffraction peaks found here agree well with those reported in the literature, where the crystal lattice of PHBV with 3HV contents below 30 mol% corresponds to the unit cell of PHB [69]. In regard to the terpolymer, the same PHB orthorhombic lattice was seen to dominate the diffractogram. This is in agreement with other studies where P(3HB-*co*-3HHx) with less than 25 mol% of HHx was seen to present a dominant PHB lattice, with slightly different *d*-spacing in the peaks than those of the homopolymer [70,71]. Therefore, all the blends presented a similar diffractogram with the characteristic peaks of the PHB lattice. However, by looking at the right diffractograms in Figure 4, the peaks associated to the (200) and (110) planes clearly shifted towards lower 2θ angles for terpolymer contents above 10 mol%. This finding suggests that a significant lower crystalline density of PHB crystals was produced in the blends with increasing the terpolymer content in excess of 10 mol%. The strongest peak observed in the neat PHBV and PHBV-containing blends at 2θ around 27° is attributed to the boron nitride used as a nucleating agent in commercial formulations [72], which was not observed in the food waste derived P(3HB-*co*-3HV-*co*-3HHx). In any case, the lower relative intensity of the peaks with respect to the amorphous halo suggests a lower crystallinity in the terpolymer than in the neat PHBV, which agrees with the DSC measurements shown above. This trend was also visible for the blends, having more crystallinity those with higher PHBV contents and being especially noticeable for the one with 50 wt% loading of the terpolymer.

### 3.5. Thermal Stability

**Figure 5:**
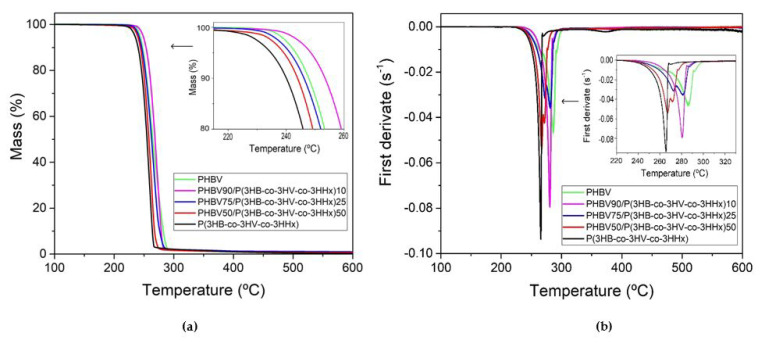
(**a**) Thermogravimetric analysis (TGA) and (**b**) first derivative (DTG) curves of the thermo-compressed films of poly(3-hydroxybutyrate-*co*-3-hydroxyvalerate) (PHBV), poly(3-hydroxybutyrate-*co*-3-hydroxyvalerate-*co*-3-hydroxyhexanoate) [P(3HB-*co*-3HV-*co*-3HHx)], and their blends.

The thermal stability of the film samples was evaluated by TGA experiments. Figure 5 shows the TGA curves of the different films and in Table 3 are gathered the most relevant parameters obtained from the TGA curves. From Figure 5a, it can be observed that the thermal degradation of both neat materials took place in a single step according to a random chain scission mechanism. This process is known to lead to a reduction in M_W_ and formation of volatile acid products such as crotonic acid [73], showing degradation temperatures (T_deg_) of 286 and 266 °C for neat PHBV and P(3HB-*co*-3HV-*co*-3HHx), respectively, in agreement with literature values [74,75]. In relation to the onset-degradation temperature (T_onset_), defined as the temperature at 5% weight loss, the terpolymer showed lower stability compared to PHBV, particularly a reduction from 243 to 235 °C [12]. The blend films presented a T_onset_ in the range of 239–243 °C, decreasing with an increase of the P(3HB-*co*-3HV-*co*-3HHx) content. As it can be seen in Figure 5b, their T_deg_ occurred in the range of 281–286 °C for the samples with lower P(3HB-*co*-3HV-*co*-3HHx) content, that is, 10 and 25 wt%, and approximately at 267 °C for the 50/50 blend. Thus, the thermal stability of the melt-mixed blends was slightly reduced with increasing the P(3HB-*co*-3HV-*co*-3HHx) content compared with PHBV, showing lower T_onset_ and T_deg_ values. This decrease in the thermal resistance can be attributed to the impurities that remained in the terpolyester and could not be removed during the purification process. In this sense, it has been reported that the presence of fermentation residues in PHA can accelerate its thermal degradation mechanisms [76]. Finally, the amount of residual mass was below 1% for all the samples, showing the neat P(3HB-*co*-3HV-*co*-3HHx) the lowest value, that is, 0.2%. This difference in the residual mass of the commercial polymer with respect to the terpolymer can be attributed to the presence of the inorganic nucleating agents, as previously observed by SEM and also confirmed by WAXD.

### 3.6. Mechanical Properties

**Figure 6:**
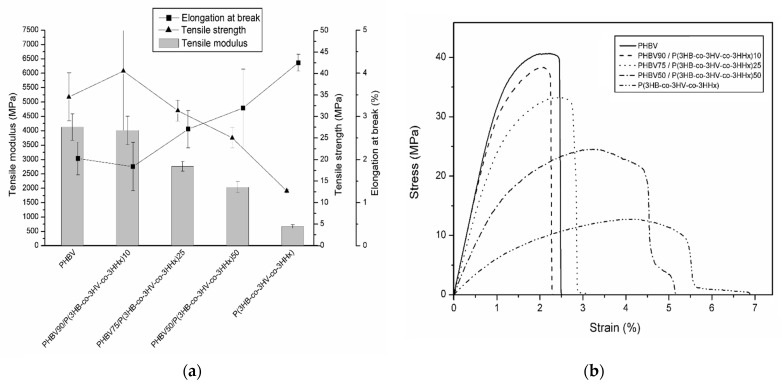
(**a**) Mechanical properties of the thermo-compressed films of poly(3-hydroxybutyrate-*co*-3-hydroxyvalerate) (PHBV), poly(3-hydroxybutyrate-*co*-3-hydroxyvalerate-*co*-3-hydroxyhexanoate) [P(3HB-*co*-3HV-*co*-3HHx)], and their blends in terms of: Tensile modulus, tensile strength, and elongation at break. (**b**) Their corresponding tensile stress–strain curves.

Figure 6 gathers the results of the tensile test results conducted on the thermo-compressed films of PHBV and P(3HB-*co*-3HV-*co*-3HHx). Figure 6a plots the main mechanical parameters obtained in the tests, while Figure 6b presents a representative stress–strain curve as obtained from the tensile tests for each of the compositions studied.

From Figure 6a, it can be seen that PHBV was a stiff material, presenting an elastic modulus at room temperature above 4 GPa and a tensile strength of approximately 35 MPa. However, the neat PHBV also exhibited a brittle behavior with an elongation-at-break value of around 2%, which occurred prior to yielding, as it can be observed in Figure 6b. The intrinsic brittleness of PHBV has been widely reported in the scientific literature and ascribed to its crystalline nature [77]. As oppose to this, P(3HB-*co*-3HV-*co*-3HHx) presented much lower elastic modulus and tensile strength than PHBV, while it also exhibited a lower brittle fracture after yielding with an elongation at break above 4%. This behavior is in accordance with previous works [12]. For instance, Bhubalan et al. [19] reported that, due to interference with the crystallization process, the higher the 3HHx fraction in the terpolymer, the higher the increase in flexibility. As it can be seen in Figure 6a, the mechanical properties of the blend films were in between those of the neat PHAs. This result means that a good interaction between both materials was achieved [78]. Thus, the tensile modulus of the blends ranged from almost 4 GPa, for the composition having a 10 wt% of terpolymer, to 2 GPa for the 50/50 blend. With respect to the tensile strength, a similar decreasing trend with the incorporation of the terpolymer was seen. Regarding ductility, an increase in the elongation at break with increasing terpolymer content was observed. This change in the mechanical response, i.e., from a rigid but fragile to a more ductile behavior, of the PHBV after melt mixing with P(3HB-*co*-3HV-*co*-3HHx), can also be spotted in the stress–strain curves in Figure 6b. From this Figure 6b, the film sample containing 50 wt% of the terpolymer was seen to exhibit a post-yielding fracture.

### 3.7. Barrier Performance

Table 4 gathers the WVP, LP, and OP values of the thermo-compressed films based on PHBV and P(3HB-*co*-3HV-*co*-3HHx). The barrier properties to gases and vapors of packaging materials play a key role in food quality and shelf-life extension aspects. The neat PHBV film presented good barrier properties to both vapors and the gas with values of 1.2 × 10^−15^ kg·m·m^−2^·Pa^−1^·s^−1^, 1.7 × 10^−15^ kg·m·m^−2^·Pa^−1^·s^−1^, and 1.6 × 10^−19^ m^3^·m·m^−2^·Pa^−1^·s^−1^ for WVP, LP, and OP, respectively. On the contrary and as expected, the barrier performance of the P(3HB-*co*-3HV-*co*-3HHx) film was significantly lower, especially to the aroma component, with values of 7.3 × 10^−15^ kg·m·m^−2^·Pa^−1^·s^−1^, 18.4 × 10^−15^ kg·m·m^−2^·Pa^−1^·s^−1^, and 5.2 × 10^−19^ m^3^·m·m^−2^·Pa^−1^·s^−1^ for WVP, LP, and OP, respectively. In relation to the blends, it can be observed that these films presented a barrier performance in between the pristine polymers. In particular, the values of permeability ranged between 1.3–3.4 × 10^−15^ kg·m·m^−2^·Pa^−1^·s^−1^, 1.8–3.7 × 10^−15^ kg·m·m^−2^·Pa^−1^·s^−1^, and 2.7–3.6 × 10^−19^ m^3^·m·m^−2^·Pa^−1^·s^−1^, respectively. In spite of the clear decrease in barrier properties, all the permeability values of the blend films were still within the same order of magnitude as the neat PHBV film.

The barrier decrease is attributed to the above-described lower crystallinity of P(3HB-*co*-3HV-*co*-3HHx) and potentially also due to the biomass impurities remaining in the terpolymer and its blends. In particular, the higher crystallinity observed for PHBV can be responsible for the lower values of OP since oxygen is a non-interacting and non-condensable permeant which permeability is driven by diffusion. As a result, the higher the crystallinity, the higher the material density and the lower the fraction of the amorphous phase, resulting in lower free volume and higher tortuosity for the gas molecules to diffuse through. Limonene, which is known to be a strong plasticizer for PHAs and highly sorbed into the amorphous regions of this biopolymer, showed values of nearly 13 wt% of uptake in solvent-cast films [79]. In comparison to PHB films also prepared by compression molding, similar WVP and LP values as the here-obtained PHBV film were reported, that is, 1.8 and 2.0 × 10^−15^ kg·m·m^−2^·Pa^−1^·s^−1^, respectively [80]. In terms of OP, similar values were also obtained, that is, 2.2 × 10^−19^ m^3^·m·m^−2^·Pa^−1^·s^−1^ [81]. For compression-molded PHBV films with 12 mol% 3HV, the values for WVP, LP, and OP were higher than the ones obtained here, that is, 6.9 × 10^−15^ kg·m·m^−2^·Pa^−1^·s^−1^, 1.9 × 10^−13^ kg·m·m^−2^·Pa^−1^·s^−1^, and 15.7 × 10^−19^ m^3^·m·m^−2^·Pa^−1^·s^−1^, respectively [81].

Furthermore, barrier properties within the same order of magnitude as the ones measured in this study, were previously reported for other PHA blends. For example, Martinez-Abad [49] reported no significant changes in water and limonene permeabilities when blending commercial PHBV with unpurified food waste derived PHBV at contents up to 15 wt%, above which there was a slight decrease. More similar barrier results to the ones measured here were found when commercial PHB was blended with PHBV obtained using MMC, where a lower barrier performance was observed with respect to the neat homopolyester. Thus, the WPV and LP values increased from 1.8 and 1.9 × 10^−15^ kg·m·m^−2^·Pa^−1^·s^−1^, for neat PHB, to 7.5 and 5.0 × 10^−15^ kg·m·m^−2^·Pa^−1^·s^−1^, for the blend obtained with the highest food waste derived PHBV content, that is, 50 wt% [40]. Finally, the barrier values obtained here are in the same range of those of polyethylene terephthalate (PET) films, with WVP and OP values of 2.3 × 10^−15^ kg·m·m^−2^·Pa^−1^·s^−1^ and 1.4 × 10^−19^ m^3^·m·m^−2^·Pa^−1^·s^−1^, respectively [82]. As expected, the blends also presented higher barrier in terms of OP than low-density polyethylene (LDPE) films, with an OP value of 2.15 × 10^−17^ m^3^·m·m^−2^·Pa^−1^·s^−1^, but, of course, lower than the high-barrier ethylene–vinyl alcohol copolymer (EVOH), with OP at 0.77 × 10^−21^ m^3^·m·m^−2^·Pa^−1^·s^−1^ [82]. Moreover, the present PHA blends showed higher barrier properties than other biopolyesters. For example, PLA and PBAT films prepared by thermo-compression presented values of 12.3 and 33.1 × 10^−15^ kg·m·m^−2^·Pa^−1^·s^−1^, for WVP, and 3.3 and 72.6 × 10^−15^ kg·m·m^−2^·Pa^−1^·s^−1^, for LP, respectively [83]. Films of these two biopolymers showed also higher OP values, that is, 2.2 and 9.1 × 10^−18^ m^3^·m·m^−2^·Pa^−1^·s^−1^, respectively [83].

## 4. Conclusions

A new PHA terpolymer, P(3HB-*co*-3HV-*co*-3HHx), was produced from MMCs fed with fruit pulp biowaste with a comonomer content of ca. 68 mol% 3HB, 17 mol% 3HV, and 15 mol% 3HHx. The terpolymer was extracted and purified using a previously developed one-step method and, thereafter, melt-mixed in contents of up to 50 wt% with commercial PHBV. The morphological analysis revealed good interpolymer miscibility and good optical properties, in spite of the presence of remnant impurities. From the DSC and WAXS results, the lateral molecular order and density were inferred to be reduced, but the crystalline morphology remained that of the PHB crystals across composition. The thermal stability of the blends was not substantially affected by the incorporation of the food waste derived terpolymer. On the other hand, the materials were seen to be more flexible than the neat rigid PHBV film as a result of the plasticizing effect brought in by the terpolymer. The permeability to water and limonene vapors and oxygen gas was reduced in the blends, particularly for limonene, but remained within the same order of magnitude.

Therefore, this research study demonstrates further the potential of PHA blends that make use of potentially lower cost PHAs derived from Circular Bioeconomy strategies to constitute novel packaging materials, which will profit from the more sustainable organic recycling end-of-life scenario. In particular, the PHA blends prepared herein can be used, depending on composition, to constitute rigid or semirigid packaging articles, such as injection-molded and thermoformed monolayer articles, and also in disposables, such as plates and trays, which can be organic recycled in industrial and home composting or, even, that will be able to biodegrade in soil or marine environments. Future studies will focus on the development of novel polymers with further enhanced flexibility by increasing the comonomer content, and in alternative purification methodologies that can achieve higher purity using water-based systems.

## Figures and Tables

**Table 1:** Set of formulations prepared according to the weight content (wt%) of poly(3-hydroxybutyrate-*co*-3-hydroxyvalerate) (PHBV) and poly(3-hydroxybutyrate-*co*-3-hydroxyvalerate-*co*-3-hydroxyhexanoate) [P(3HB-*co*-3HV-*co*-3HHx)].

Sample	PHBV (wt%)	P(3HB-co-3HV-co-3HHx) (wt%)
PHBV	100	0
PHBV90/P(3HB-*co*-3HV-*co*-3HHx)10	90	10
PHBV75/P(3HB-*co*-3HV-*co*-3HHx)25	75	25
PHBV50/P(3HB-*co*-3HV-*co*-3HHx)50	50	50
P(3HB-*co*-3HV-*co*-3HHx)	0	100

**Table 2:** Color parameters (a*^,^ b*, L*, and ∆E*) and transparency (T) and opacity (O) values of the thermo-compressed films of poly(3-hydroxybutyrate-*co*-3-hydroxyvalerate) (PHBV), poly(3-hydroxybutyrate-*co*-3-hydroxyvalerate-*co*-3-hydroxyhexanoate) [P(3HB-*co*-3HV-*co*-3HHx)], and their blends.

Film	a*	b*	L*	ΔE*	T	O
PHBV	-0.46 ± 0.07 ^a,d^	5.58 ± 0.20 ^a^	88.25 ± 0.10 ^a^	-	9.55 ± 0.35 ^a^	0.11 ± 0.07 ^a^
PHBV90/P(3HB-*co*-3HV-*co*-3HHx)10	-0.56 ± 0.03 ^a,c^	6.92 ± 0.30 ^b^	87.52 ± 0.14 ^a^	1.53 ± 0.18 ^a^	8.10 ± 0.42 ^b^	0.10 ± 0.03 ^a^
PHBV75/P(3HB-*co*-3HV-*co*-3HHx)25	-0.19 ± 0.02 ^b^	3.70 ± 0.09 ^c^	88.59 ± 0.30 ^a^	1.93 ± 0.11 ^a^	9.29 ± 0.38 ^a,c^	0.11 ± 0.04 ^a^
PHBV50/P(3HB-*co*-3HV-*co*-3HHx)50	-0.69 ± 0.04 ^c^	4.72 ± 0.07 ^d^	88.29 ± 0.22 ^a^	0.89 ± 0.07 ^c^	8.64 ± 0.33 ^b,c^	0.11 ± 0.04 ^a^
P(3HB-*co*-3HV-*co*-3HHx)	-0.36 ± 0.02 ^d^	7.84 ± 0.10 ^e^	83.83 ± 0.52 ^b^	4.97 ± 0.21 ^d^	2.85 ± 0.19 ^d^	0.15 ± 0.05 ^a^

a*: Red/green coordinates (+a red, −a green); b*: Yellow/blue coordinates (+b yellow, −b blue); L*: Luminosity (+L luminous, −L dark); ΔE*: Color differences; T: Transparency; O: Opacity.^a–e^ Different letters in the same column indicate a significant difference among the samples (*p* < 0.05).

**Table 3:** Main thermogravimetric analysis (TGA) parameters of the thermo-compressed films of poly(3-hydroxybutyrate-*co*-3-hydroxyvalerate) (PHBV), poly(3-hydroxybutyrate-*co*-3-hydroxyvalerate-*co*-3-hydroxyhexanoate) [P(3HB-*co*-3HV-*co*-3HHx)], and their blends in terms of: Onset temperature of degradation (T_5%_), degradation temperature (T_deg_), mass loss at T_deg_, and residual mass at 800 °C.

Film	T_5%_ (°C)	T_deg_ (°C)	Mass Loss (%)	Residual Mass (%)
PHBV	243.3 ± 1.2 ^a^	286.2 ± 0.9 ^a^	95.4 ± 0.8 ^a^	1.0 ± 0.2 ^a^
PHBV90/P(3HB-*co*-3HV-*co*-3HHx)10	242.5 ± 1.5 ^a^	280.5 ± 1.2 ^b^	94.6 ± 1.0 ^a^	0.8 ± 0.1 ^a^
PHBV75/P(3HB-*co*-3HV-*co*-3HHx)25	241.4 ± 1.8 ^a,b^	281.1 ± 1.3 ^b^	95.3 ± 0.9 ^a^	0.9 ± 0.2 ^a^
PHBV50/P(3HB-*co*-3HV-*co*-3HHx)50	239.2 ± 1.3 ^b^	267.1 ± 1.1 ^c^	88.3 ± 1.2 ^b^	0.7 ± 0.2 ^a^
P(3HB-*co*-3HV-*co*-3HHx)	234.8 ± 1.2 ^c^	265.5 ± 1.6 ^c^	94.5 ± 1.1 ^a^	0.2 ± 0.1 ^b^

^a–c^ Different letters in the same column indicate a significant difference among the samples (*p* < 0.05).

**Table 4:** Water vapor permeability (WVP), _D_-limonene permeability (LP), and oxygen permeability (OP) of the thermo-compressed films of poly(3-hydroxybutyrate-*co*-3-hydroxyvalerate) (PHBV), poly(3-hydroxybutyrate-*co*-3-hydroxyvalerate-*co*-3-hydroxyhexanoate) [P(3HB-*co*-3HV-*co*-3HHx)], and their blends.

Film	WVP × 10 ^15^ (kg·m/m^2^·Pa·s)	LP × 10 ^15^ (kg·m/m^2^·Pa·s)	OP × 10 ^19^ (m^3^·m/m^2^·Pa·s)
PHBV	1.19 ± 0.08 ^a^	1.73 ± 0.22 ^a^	1.60 ± 0.24 ^a^
PHBV90/P(3HB-*co*-3HV-*co*-3HHx)10	1.27 ± 0.14 ^a^	1.80 ± 0.15 ^a^	2.69 ± 0.38 ^b^
PHBV75/P(3HB-*co*-3HV-*co*-3HHx)25	2.42 ± 0.41 ^b^	2.03 ± 0.21 ^a^	3.34 ± 0.51 ^b^
PHBV50/P(3HB-*co*-3HV-*co*-3HHx)50	3.39 ± 0.33 ^c^	3.65 ± 0.44 ^b^	3.61 ± 0.47 ^b,c^
P(3HB-*co*-3HV-*co*-3HHx)	7.29 ± 0.89 ^d^	18.4 ± 6.9 ^c^	5.16 ± 1.05 ^c^

^a–d^ Different letters in the same column indicate a significant difference among the samples (*p* < 0.05).

## Data Availability

Not applicable.

## Supplementary Materials

The following are available online at https://www.mdpi.com/article/10.3390/polym13071155/s1, Table S1: Differential scanning calorimetry (DSC) parameters of the thermo-compressed films made of poly(3-hydroxybutyrate-*co*-3-hydroxyvalerate) (PHBV) and poly(3-hydroxybutyrate-*co*-3-hydroxyvalerate-*co*-3-hydroxyhexanoate) [P(3HB-*co*-3HV-*co*-3HHx)] in terms of: Glass transition temperature (T_g_), melting temperature (T_m_), enthalpy of melting (∆H_m_), crystallization temperature (T_c_), enthalpy of crystallization (ΔH_c_), cold crystallization temperature (T_cc_), and enthalpy of the cold crystallization (∆H_cc_).

## References

[1] Barnes D.K.A., Galgani F., Thompson R.C., Barlaz M. (2009). Accumulation and fragmentation of plastic debris in global environments. Philos. Trans. R. Soc. B Biol. Sci..

[2] Alfei S., Schito A.M., Zuccari G. (2021). Biodegradable and Compostable Shopping Bags under Investigation by FTIR Spectroscopy. Appl. Sci..

[3] Torres-Giner S., Figueroa-Lopez K.J., Melendez-Rodriguez B., Prieto C., Pardo-Figuerez M., Lagaron J.M. (2021). Emerging Trends in Biopolymers for Food Packaging. Sustainable Food Packaging Technology.

[4] REHM B.H.A. (2003). Polyester synthases: Natural catalysts for plastics. Biochem. J..

[5] Dobrucka R. (2019). Bioplastic packaging materials in circular economy. Logforum.

[6] Winnacker M. (2019). Polyhydroxyalkanoates: Recent Advances in Their Synthesis and Applications. Eur. J. Lipid Sci. Technol..

[7] Madison L.L., Huisman G.W. (1999). Metabolic engineering of poly(3-hydroxyalkanoates): From DNA to plastic. Microbiol. Mol. Biol. Rev..

[8] Mitomo H., Barham P.J., Keller A. (1987). Crystallization and Morphology of Poly(β-hydroxybutyrate) and Its Copolymer. Polym. J..

[9] Chan C.M., Vandi L.-J., Pratt S., Halley P., Ma Y., Chen G.-Q., Richardson D., Werker A., Laycock B. (2019). Understanding the effect of copolymer content on the processability and mechanical properties of polyhydroxyalkanoate (PHA)/wood composites. Compos. Part A Appl. Sci. Manuf..

[10] Savenkova L., Gercberga Z., Bibers I., Kalnin M. (2000). Effect of 3-hydroxy valerate content on some physical and mechanical properties of polyhydroxyalkanoates produced by *Azotobacter chroococcum*. Process Biochem..

[11] Martínez-Sanz M., Villano M., Oliveira C., Albuquerque M.G.E., Majone M., Reis M., Lopez-Rubio A., Lagaron J.M. (2014). Characterization of polyhydroxyalkanoates synthesized from microbial mixed cultures and of their nanobiocomposites with bacterial cellulose nanowhiskers. New Biotechnol..

[12] Zhao W., Chen G.-Q. (2007). Production and characterization of terpolyester poly(3-hydroxybutyrate-co-3-hydroxyvalerate-co-3-hydroxyhexanoate) by recombinant Aeromonas hydrophila 4AK4 harboring genes phaAB. Process Biochem..

[13] Ye H.-M., Wang Z., Wang H.-H., Chen G.-Q., Xu J. (2010). Different thermal behaviors of microbial polyesters poly(3-hydroxybutyrate-co-3-hydroxyvalerate-co-3-hydroxyhexanoate) and poly(3-hydroxybutyrate-co-3-hydroxyhexanoate). Polymer.

[14] Liang Y.-S., Zhao W., Chen G.-Q. (2008). Study on the biocompatibility of novel terpolyester poly(3-hydroxybutyrate-co-3-hydroxyvalerate-co-3-hydroxyhexanoate). J. Biomed. Mater. Res. Part A.

[15] Ji Y., Li X.-T., Chen G.-Q. (2008). Interactions between a poly(3-hydroxybutyrate-co-3-hydroxyvalerate-co-3-hydroxyhexanoate) terpolyester and human keratinocytes. Biomaterials.

[16] Brandl H., Knee E.J., Fuller R.C., Gross R.A., Lenz R.W. (1989). Ability of the phototrophic bacterium *Rhodospirillum rubrum* to produce various poly (β-hydroxyalkanoates): Potential sources for biodegradable polyesters. Int. J. Biol. Macromol..

[17] Haywood G.W., Anderson A.J., Roger Williams D., Dawes E.A., Ewing D.F. (1991). Accumulation of a poly(hydroxyalkanoate) copolymer containing primarily 3-hydroxyvalerate from simple carbohydrate substrates by *Rhodococcus* sp. NCIMB 40126. Int. J. Biol. Macromol..

[18] Park S.J., Ahn W.S., Green P.R., Lee S.Y. (2001). Biosynthesis of poly(3-hydroxybutyrate-co-3-hydroxyvalerate-co-3-hydroxyhexanoate) by metabolically engineered *Escherichia coli* strains. Biotechnol. Bioeng..

[19] Bhubalan K., Rathi D.-N., Abe H., Iwata T., Sudesh K. (2010). Improved synthesis of P(3HB-co-3HV-co-3HHx) terpolymers by mutant Cupriavidus necator using the PHA synthase gene of *Chromobacterium* sp. USM2 with high affinity towards 3HV. Polym. Degrad. Stab..

[20] Jung H.-R., Jeon J.-M., Yi D.-H., Song H.-S., Yang S.-Y., Choi T.-R., Bhatia S.K., Yoon J.-J., Kim Y.-G., Brigham C.J. (2019). Poly(3-hydroxybutyrate-co-3-hydroxyvalerate-co-3-hydroxyhexanoate) terpolymer production from volatile fatty acids using engineered *Ralstonia eutropha*. Int. J. Biol. Macromol..

[21] Reiser S.E., Mitsky T.A., Gruys K.J. (2000). Characterization and cloning of an (R)-specific trans-2,3-enoylacyl-CoA hydratase from Rhodospirillum rubrum and use of this enzyme for PHA production in Escherichia coli. Appl. Microbiol. Biotechnol..

[22] Zhang H.-F., Ma L., Wang Z.-H., Chen G.-Q. (2009). Biosynthesis and characterization of 3-hydroxyalkanoate terpolyesters with adjustable properties by *Aeromonas hydrophila*. Biotechnol. Bioeng..

[23] Choi J.I., Lee S.Y. (1997). Process analysis and economic evaluation for poly(3-hydroxybutyrate) production by fermentation. Bioprocess Eng..

[24] RamKumar Pandian S., Deepak V., Kalishwaralal K., Rameshkumar N., Jeyaraj M., Gurunathan S. (2010). Optimization and fed-batch production of PHB utilizing dairy waste and sea water as nutrient sources by *Bacillus megaterium* SRKP-3. Bioresour. Technol..

[25] Koller M., Bona R., Braunegg G., Hermann C., Horvat P., Kroutil M., Martinz J., Neto J., Pereira L., Varila P. (2005). Production of Polyhydroxyalkanoates from Agricultural Waste and Surplus Materials. Biomacromolecules.

[26] Ali Hassan M., Shirai Y., Kusubayashi N., Ismail Abdul Karim M., Nakanishi K., Hashimoto K. (1996). Effect of organic acid profiles during anaerobic treatment of palm oil mill effluent on the production of polyhydroxyalkanoates by *Rhodobacter sphaeroides*. J. Ferment. Bioeng..

[27] Dionisi D., Carucci G., Papini M.P., Riccardi C., Majone M., Carrasco F. (2005). Olive oil mill effluents as a feedstock for production of biodegradable polymers. Water Res..

[28] Tripathi A.D., Yadav A., Jha A., Srivastava S.K. (2012). Utilizing of Sugar Refinery Waste (Cane Molasses) for Production of Bio-Plastic Under Submerged Fermentation Process. J. Polym. Environ..

[29] Bengtsson S., Werker A., Christensson M., Welander T. (2008). Production of polyhydroxyalkanoates by activated sludge treating a paper mill wastewater. Bioresour. Technol..

[30] Duque A.F., Oliveira C.S.S., Carmo I.T.D., Gouveia A.R., Pardelha F., Ramos A.M., Reis M.A.M. (2014). Response of a three-stage process for PHA production by mixed microbial cultures to feedstock shift: Impact on polymer composition. New Biotechnol..

[31] Bosco F., Chiampo F. (2010). Production of polyhydroxyalcanoates (PHAs) using milk whey and dairy wastewater activated sludge: Production of bioplastics using dairy residues. J. Biosci. Bioeng..

[32] Arumugam A., Anudakshaini T.S., Shruthi R., Jeyavishnu K., Sundarra Harini S., Sharad J.S. (2019). Low-cost production of PHA using cashew apple (*Anacardium occidentale* L.) juice as potential substrate: Optimization and characterization. Biomass Convers. Biorefinery.

[33] Khumwanich P., Napathorn S.C., Suwannasilp B.B. (2014). Polyhydroxyalkanoate Production with a Feast/Famine Feeding Regime Using Sludge from Wastewater Treatment Plants of the Food and Beverage Industry. J. Biobased Mater. Bioenergy.

[34] Papa G., Pepè Sciarria T., Carrara A., Scaglia B., D’Imporzano G., Adani F. (2020). Implementing polyhydroxyalkanoates production to anaerobic digestion of organic fraction of municipal solid waste to diversify products and increase total energy recovery. Bioresour. Technol..

[35] Babu R.P., O’Connor K., Seeram R. (2013). Current progress on bio-based polymers and their future trends. Prog. Biomater..

[36] Zembouai I., Bruzaud S., Kaci M., Benhamida A., Corre Y.-M., Grohens Y., Lopez-Cuesta J.-M. (2014). Synergistic effect of compatibilizer and cloisite 30B on the functional properties of poly(3-hydroxybutyrate-co-3-hydroxyvalerate)/polylactide blends. Polym. Eng. Sci..

[37] Nishida M., Tanaka T., Hayakawa Y., Ogura T., Ito Y., Nishida M. (2019). Multi-scale instrumental analyses of plasticized polyhydroxyalkanoates (PHA) blended with polycaprolactone (PCL) and the effects of crosslinkers and graft copolymers. Rsc Adv..

[38] Sánchez-Safont E.L., Aldureid A., Lagarón J.M., Gamez-Perez J., Cabedo L. (2020). Effect of the Purification Treatment on the Valorization of Natural Cellulosic Residues as Fillers in PHB-Based Composites for Short Shelf Life Applications. Waste Biomass Valorization.

[39] Torres-Tello E.V., Robledo-Ortíz J.R., González-García Y., Pérez-Fonseca A.A., Jasso-Gastinel C.F., Mendizábal E. (2017). Effect of agave fiber content in the thermal and mechanical properties of green composites based on polyhydroxybutyrate or poly(hydroxybutyrate-co-hydroxyvalerate). Ind. Crop. Prod..

[40] Melendez-Rodriguez B., Torres-Giner S., Aldureid A., Cabedo L., Lagaron J.M. (2019). Reactive Melt Mixing of Poly(3-Hydroxybutyrate)/Rice Husk Flour Composites with Purified Biosustainably Produced Poly(3-Hydroxybutyrate-*co*-3-Hydroxyvalerate). Materials.

[41] Sarasini F., Luzi F., Dominici F., Maffei G., Iannone A., Zuorro A., Lavecchia R., Torre L., Carbonell-Verdu A., Balart R. (2018). Effect of Different Compatibilizers on Sustainable Composites Based on a PHBV/PBAT Matrix Filled with Coffee Silverskin. Polymers.

[42] Furukawa T., Sato H., Murakami R., Zhang J., Noda I., Ochiai S., Ozaki Y. (2007). Comparison of miscibility and structure of poly(3-hydroxybutyrate-co-3-hydroxyhexanoate)/poly(l-lactic acid) blends with those of poly(3-hydroxybutyrate)/poly(l-lactic acid) blends studied by wide angle X-ray diffraction, differential scanning calorimetry, and FTIR microspectroscopy. Polymer.

[43] Peng S., An Y., Chen C., Fei B., Zhuang Y., Dong L. (2003). Miscibility and crystallization behavior of poly(3-hydroxyvalerate-co-3-hydroxyvalerate)/ poly(propylene carbonate) blends. J. Appl. Polym. Sci..

[44] Choe S., Cha Y.-J., Lee H.-S., Yoon J.S., Choi H.J. (1995). Miscibility of poly(3-hydroxybutyrate-co-3-hydroxyvalerate) and poly(vinyl chloride) blends. Polymer.

[45] Zembouai I., Bruzaud S., Kaci M., Benhamida A., Corre Y.-M., Grohens Y., Taguet A., Lopez-Cuesta J.-M. (2014). Poly(3-Hydroxybutyrate-co-3-Hydroxyvalerate)/Polylactide Blends: Thermal Stability, Flammability and Thermo-Mechanical Behavior. J. Polym. Environ..

[46] Nerkar M., Ramsay J.A., Ramsay B.A., Kontopoulou M. (2014). Melt Compounded Blends of Short and Medium Chain-Length Poly-3-hydroxyalkanoates. J. Polym. Environ..

[47] Verma D., Goh K.L., Jawaid M., Bouhfid R., Kacem Qaiss A.E. (2019). Chapter 11–Functionalized Graphene-Based Nanocomposites for Energy Applications. Functionalized Graphene Nanocomposites and Their Derivatives.

[48] Koyama T., Tanoue S., Iemoto Y., Maekawa T., Unryu T. (2009). Melt compounding of various polymers with organoclay by shear flow. Polym. Compos..

[49] Martínez-Abad A., Cabedo L., Oliveira C.S.S., Hilliou L., Reis M., Lagarón J.M. (2016). Characterization of polyhydroxyalkanoate blends incorporating unpurified biosustainably produced poly(3-hydroxybutyrate-co-3-hydroxyvalerate). J. Appl. Polym. Sci..

[50] Lanham A.B., Ricardo A.R., Albuquerque M.G.E., Pardelha F., Carvalheira M., Coma M., Fradinho J., Carvalho G., Oehmen A., Reis M.A.M. (2013). Determination of the extraction kinetics for the quantification of polyhydroxyalkanoate monomers in mixed microbial systems. Process Biochem..

[51] Rebocho A.T., Pereira J.R., Neves L.A., Alves V.D., Sevrin C., Grandfils C., Freitas F., Reis M.A.M. (2020). Preparation and Characterization of Films Based on a Natural P(3HB)/mcl-PHA Blend Obtained through the Co-culture of Cupriavidus Necator and Pseudomonas Citronellolis in Apple Pulp Waste. Bioengineering.

[52] Fiorese M.L., Freitas F., Pais J., Ramos A.M., de Aragão G.M.F., Reis M.A.M. (2009). Recovery of polyhydroxybutyrate (PHB) from Cupriavidus necator biomass by solvent extraction with 1,2-propylene carbonate. Eng. Life Sci..

[53] Shiku Y., Yuca Hamaguchi P., Benjakul S., Visessanguan W., Tanaka M. (2004). Effect of surimi quality on properties of edible films based on Alaska pollack. Food Chem..

[54] Kanatt S., Rao M.S., Chawla S., Sharma A. (2012). Active chitosan–polyvinyl alcohol films with natural extract. Food Hydrocoll..

[55] Arfat Y.A., Ahmed J., Hiremath N., Auras R., Joseph A. (2017). Thermo-mechanical, rheological, structural and antimicrobial properties of bionanocomposite films based on fish skin gelatin and silver-copper nanoparticles. Food Hydrocoll..

[56] Mokrzycki W., Tatol M. (2011). Color difference Delta E–A survey. Mach. Graph. Vis..

[57] Ivorra-Martinez J., Quiles-Carrillo L., Boronat T., Torres-Giner S.A., Covas J. (2020). Assessment of the Mechanical and Thermal Properties of Injection-Molded Poly(3-hydroxybutyrate-co-3-hydroxyhexanoate)/Hydroxyapatite Nanoparticles Parts for Use in Bone Tissue Engineering. Polymers.

[58] Gómez-Guillén M.C., Ihl M., Bifani V., Silva A., Montero P. (2007). Edible films made from tuna-fish gelatin with antioxidant extracts of two different murta ecotypes leaves (*Ugni molinae* Turcz). Food Hydrocoll..

[59] Doi Y., Kitamura S., Abe H. (1995). Microbial Synthesis and Characterization of Poly(3-hydroxybutyrate-co-3-hydroxyhexanoate). Macromolecules.

[60] Ivorra-Martinez J., Manuel-Mañogil J., Boronat T., Sanchez-Nacher L., Balart R., Quiles-Carrillo L. (2020). Development and Characterization of Sustainable Composites from Bacterial Polyester Poly(3-Hydroxybutyrate-co-3-hydroxyhexanoate) and Almond Shell Flour by Reactive Extrusion with Oligomers of Lactic Acid. Polymers.

[61] Vahabi H., Michely L., Moradkhani G., Akbari V., Cochez M., Vagner C., Renard E., Saeb M.R., Langlois V. (2019). Thermal Stability and Flammability Behavior of Poly(3-hydroxybutyrate) (PHB) Based Composites. Mater..

[62] Vandewijngaarden J., Wauters R., Murariu M., Dubois P., Carleer R., Yperman J., D’Haen J., Ruttens B., Schreurs S., Lepot N. (2016). Poly(3-hydroxybutyrate-co-3-hydroxyhexanoate)/Organomodified Montmorillonite Nanocomposites for Potential Food Packaging Applications. J. Polym. Environ..

[63] Cai H., Qiu Z. (2009). Effect of comonomer content on the crystallization kinetics and morphology of biodegradable poly(3-hydroxybutyrate-co-3-hydroxyhexanoate). Phys. Chem. Chem. Phys..

[64] Kai Z., Ying D., Guo-Qiang C. (2003). Effects of surface morphology on the biocompatibility of polyhydroxyalkanoates. Biochem. Eng. J..

[65] Qu X.-H., Wu Q., Liang J., Zou B., Chen G.-Q. (2006). Effect of 3-hydroxyhexanoate content in poly(3-hydroxybutyrate-co-3-hydroxyhexanoate) on in vitro growth and differentiation of smooth muscle cells. Biomaterials.

[66] Rojas-Lema S., Quiles-Carrillo L., Garcia-Garcia D., Melendez-Rodriguez B., Balart R., Torres-Giner S. (2020). Tailoring the Properties of Thermo-Compressed Polylactide Films for Food Packaging Applications by Individual and Combined Additions of Lactic Acid Oligomer and Halloysite Nanotubes. Molecules.

[67] Sato H., Suttiwijitpukdee N., Hashimoto T., Ozaki Y. (2012). Simultaneous Synchrotron SAXS/WAXD Study of Composition Fluctuations, Cold-Crystallization, and Melting in Biodegradable Polymer Blends of Cellulose Acetate Butyrate and Poly(3-hydroxybutyrate). Macromolecules.

[68] Panaitescu D.M., Nicolae C.A., Frone A.N., Chiulan I., Stanescu P.O., Draghici C., Iorga M., Mihailescu M. (2017). Plasticized poly(3-hydroxybutyrate) with improved melt processing and balanced properties. J. Appl. Polym. Sci..

[69] Skrbić Z., Divjaković V. (1996). Temperature influence on changes of parameters of the unit cell of biopolymer PHB. Polymer.

[70] Sato H., Nakamura M., Padermshoke A., Yamaguchi H., Terauchi H., Ekgasit S., Noda I., Ozaki Y. (2004). Thermal Behavior and Molecular Interaction of Poly(3-hydroxybutyrate-co-3-hydroxyhexanoate) Studied by Wide-Angle X-ray Diffraction. Macromolecules.

[71] Xie Y., Noda I., Akpalu Y.A. (2008). Influence of cooling rate on the thermal behavior and solid-state morphologies of polyhydroxyalkanoates. J. Appl. Polym. Sci..

[72] Öner M., Kızıl G., Keskin G., Pochat-Bohatier C., Bechelany M. (2018). The Effect of Boron Nitride on the Thermal and Mechanical Properties of Poly(3-hydroxybutyrate-co-3-hydroxyvalerate). Nanomater..

[73] Bhardwaj R., Mohanty A.K., Drzal L.T., Pourboghrat F., Misra M. (2006). Renewable Resource-Based Green Composites from Recycled Cellulose Fiber and Poly(3-hydroxybutyrate-co-3-hydroxyvalerate) Bioplastic. Biomacromolecules.

[74] Ferreira B.M.P., Zavaglia C.A.C., Duek E.A.R. (2001). Films of poly (L-lactic acid)/poly(hydroxybutyrate-co-hydroxyvalerate) blends: In vitro degradation. Mater. Res..

[75] Hu Y.-J., Wei X., Zhao W., Liu Y.-S., Chen G.-Q. (2009). Biocompatibility of poly(3-hydroxybutyrate-co-3-hydroxyvalerate-co-3-hydroxyhexanoate) with bone marrow mesenchymal stem cells. Acta Biomater..

[76] Hablot E., Bordes P., Pollet E., Avérous L. (2008). Thermal and thermo-mechanical degradation of poly(3-hydroxybutyrate)-based multiphase systems. Polym. Degrad. Stab..

[77] Sanchez-Safont E.L., Cabedo L., Gamez-Perez J. (2021). Cellulose-Reinforced Biocomposites Based on PHB and PHBV for Food Packaging Applications. Sustainable Food Packaging Technology.

[78] Martínez-Abad A., González-Ausejo J., Lagarón J.M., Cabedo L. (2016). Biodegradable poly(3-hydroxybutyrate-co-3-hydroxyvalerate)/thermoplastic polyurethane blends with improved mechanical and barrier performance. Polym. Degrad. Stab..

[79] Sanchez-Garcia M.D., Gimenez E., Lagaron J.M. (2008). Morphology and barrier properties of solvent cast composites of thermoplastic biopolymers and purified cellulose fibers. Carbohydr. Polym..

[80] Cherpinski A., Torres-Giner S., Cabedo L., Lagaron J.M. (2017). Post-processing optimization of electrospun submicron poly(3-hydroxybutyrate) fibers to obtain continuous films of interest in food packaging applications. Food Addit. Contam. Part A.

[81] Sanchez-Garcia M.D., Gimenez E., Lagaron J.M. (2007). Novel PET Nanocomposites of Interest in Food Packaging Applications and Comparative Barrier Performance with Biopolyester Nanocomposites. J. Plast. Film Sheeting.

[82] Lagaron J.M., Catalá R., Gavara R. (2004). Structural characteristics defining high barrier properties in polymeric materials. Mater. Sci. Technol..

[83] Quiles-Carrillo L., Montanes N., Lagaron J.M., Balart R., Torres-Giner S. (2019). In Situ Compatibilization of Biopolymer Ternary Blends by Reactive Extrusion with Low-Functionality Epoxy-Based Styrene–Acrylic Oligomer. J. Polym. Environ..

