# Supplementary material for: Blends of Poly(3-Hydroxybutyrate-co-3-Hydroxyvalerate) with Fruit Pulp Biowaste Derived Poly(3-Hydroxybutyrate-co-3-Hydroxyvalerate-co-3-Hydroxyhexanoate) for Organic Recycling Food Packaging
Source: Polymers (Basel). 2021 Apr 4;13(7):1155. doi: 10.3390/polym13071155 (PMC8038484; doi:10.3390/polym13071155)
Supplement: Supplementary file 1 [file polymers-13-01155-s001.pdf]

Supporting Information

Blends of Poly(3-hydroxybutyrate-*co*-3-hydroxyvalerate) with Fruit Pulp Biowaste Derived Poly(3-hydroxybutyrate-*co*-3-hydroxyvalerate-*co*-3-hydroxyhexanoate) for Organic Recycling Food Packaging

Beatriz Meléndez-Rodríguez[1], Sergio Torres-Giner[1ς], Maria A.M. Reis[2], Fernando Silva[2], Mariana Matos[2], Luis Cabedo[3] and José María Lagarón[1*]

[1] Novel Materials and Nanotechnology Group, Institute of Agrochemistry and Food Technology (IATA), Spanish Council for Scientific Research (CSIC), Calle Catedrático Agustín Escardino Benlloch 7, 46980 Paterna, Spain; beatriz.melendez@iata.csic.es

[2] UCIBIO-REQUIMTE-Applied Molecular Biosciences Unit, Chemistry Department, Faculty of Sciences and Technology, New University of Lisbon, 1099-085 Lisbon, Portugal; amr@fct.unl.pt; fra.silva@campus.fct.unl.pt; m.matos@campus.fct.unl.pt

[3] Polymers and Advanced Materials Group (PIMA), Universitat Jaume I (UJI), 12071 Castellón, Spain; lcabedo@uji.es

[ς] This author is currently with the Research Institute of Food Engineering for Development (IIAD), Universitat Politècnica de València (UPV), Camino de Vera s/n, 46022 Valencia, Spain; storresginer@upv.es

[*] Correspondence: lagaron@iata.csic.es; Tel.: +34-963-900-022

Table S1. DSC parameters of the thermo-compressed films made of poly(3-hydroxybutyrate-*co*-3-hydroxyvalerate) (PHBV) and poly(3-hydroxybutyrate-*co*-3-hydroxyvalerate-*co*-3-hydroxyhexanoate) [P(3HB-*co*-3HV-*co*-3HHx)] in terms of: glass transition temperature (T_g), melting temperature (T_m), enthalpy of melting (ΔH_m), crystallization temperature (T_c), enthalpy of crystallization (ΔH_c), cold crystallization temperature (T_{cc}), and enthalpy of the cold crystallization (ΔH_{cc}).

Film	First heating			Cooling				Second heating				
	T_g (°C)	T_m (°C)	ΔH_m (J/g)	T_{c1} (°C)	T_{c2} (°C)	ΔH_{c1} (J/g)	ΔH_{c2} (J/g)	T_g (°C)	T_{cc} (°C)	ΔH_{cc} (J/g)	T_m (°C)	ΔH_m (J/g)
PHBV	1.7 ± 0.2[a]	177.6 ± 3.1[a]	74.4 ± 8.0[a]	-	122.7 ± 2.1[a]	-	81.6 ± 1.7[a]	1.6 ± 0.1[a]	-	-	174.5 ± 3.7[a]	85.2 ± 6.7[a]
PHBV90 / P(3HB-*co*-3HV-*co*-3HHx)10	1.4 ± 0.3[a]	169.3 ± 2.4[b]	74.4 ± 9.4[a]	-	119.2 ± 0.3[b]	-	74.4 ± 3.6[b]	1.2 ± 0.2[a]	-	-	170.4 ± 0.1[a]	76.4 ± 3.9[a]
PHBV75 / P(3HB-*co*-3HV-*co*-3HHx)25	0.7 ± 0.2[b]	170.6 ± 0.4[b]	68.2 ± 6.2[a]	-	115.1 ± 0.1[c,d]	-	64.4 ± 2.7[c]	0.6 ± 0.1[b]	-	-	168.1 ± 0.1[b]	65.5 ± 4.3[b]
PHBV50 / P(3HB-*co*-3HV-*co*-3HHx)50	0.6 ± 0.1[b]	174.4 ± 0.9[a]	43.2 ± 5.4[b]	-	118.9 ± 2.0[b,d]	-	50.8 ± 2.3[d]	0.5 ± 0.1[b]	-	-	166.6 ± 3.3[b]	50.5 ± 2.1[c]
P(3HB-*co*-3HV-*co*-3HHx)	0.2 ± 0.1[c]	110.5 ± 1.3[c] // 173.3 ± 1.8[a,b]	33.4 ± 0.2[c]	65.2 ± 1.2	114.4 ± 3.3[d]	2.8 ± 0.4	6.9 ± 2.5[e]	0.6 ± 0.1[b]	71.5 ± 3.2	15.1 ± 1.6	119.7 ± 0.8[c] // 168.7 ± 1.4[b]	24.4 ± 1.7[d]

[a-d] Different letters in the same column indicate a significant difference among the samples ($p < 0.05$).
